# Long-term results of a digital treatment tool as an add-on to pediatric obesity lifestyle treatment: a 3-year pragmatic clinical trial

**DOI:** 10.1038/s41366-025-01738-0

**Published:** 2025-03-12

**Authors:** Emilia Hagman, Louise Lindberg, Resthie R. Putri, Andreas Drangel, Claude Marcus, Pernilla Danielsson

**Affiliations:** 1 Department of Clinical Science, Intervention and Technology, Division of Pediatrics, Karolinska Institutet, Stockholm, Sweden; 2 Evira AB, Triewaldsgränd 2, S-111 29 Stockholm, Sweden

**Keywords:** Obesity, Paediatrics, Weight management

## Abstract

**Background:**

The integration of mobile health technology with physical visits shows promising one-year treatment outcomes, but long-term evidence is lacking.

**Objectives:**

To assess three-year treatment outcomes for patients utilizing the digital treatment tool Evira in combination with physical visits, compared with standard obesity care.

**Methods:**

In a pragmatic trial, children with obesity aged 4.0–17.9 years receiving digi-physical treatment with Evira (*n* = 107) were compared with a group receiving standard treatment (*n* = 321). Evira comprises a digitless body scale for home-weighing, a mobile application, and a web-based clinic interface, enabling easy family-clinic communication and continuous visual treatment feedback.

**Results:**

At the three-year follow-up, the adjusted average change in body mass index Z-score was –0.29 [95% confidence interval: –0.40, –0.18] units in the digi-physical treatment group vs. –0.12 [–0.21, –0.03] in the standard treatment group, *p* = 0.02, and 31.8% vs. 18.7% obtained obesity remission respectively, *p* = 0.0046.

**Conclusion:**

Over a three-year period, the digi-physical treatment generated superior treatment effect and higher obesity remission rate than standard treatment.

## Introduction

Treating pediatric obesity effectively is challenging due to its chronic and multifaceted nature [1]. Typically, the effectiveness of standard evidence-based lifestyle care is limited in reducing the degree of obesity [2]. Intensive behavioral programs with long-term, frequent interactions among patients, families, and healthcare providers have proven more effective [3]. However, barriers such as limited health care resources and significant time commitments make the required frequency for successful treatment difficult to achieve. Integrating mobile health technology in pediatric lifestyle obesity treatment offers a valuable way to improve the outcome. However, mobile health comprises various technologies and family support tools, with no consensus on superiority [4]. In addition, long-term studies are scarce [4]. Promising results with the digital treatment tool Evira, have been published in a 6-month randomized feasibility study [5] and a 12-month pragmatical trial [6]. Children treated with Evira, as an add-on to standard care, achieved twice as good treatment results compared to a conventional treated group [6]. The Evira treatment approach is markedly different from other scientifically tested concepts, employing a goal-oriented strategy similar to treatments for diabetes, hypertension and asthma. Objectively measured data is utilized to graphically visualize relative weight development, creating a shared understanding of treatment progress between families and clinical staff. Additionally, the tool provides a platform for communication through written messages, and efficiently identifies patients in need of additional support. The communication strategy, anchored in motivational interviewing, emphasizes empowerment over education [7].

This trial was a continuation of our previous one-year trial [6], aiming to assess the treatment effect, non-retention rate, and obesity remission incidence with Evira over three-years.

## Methods

Patients and setting are described elsewhere [6]. In short, the digi-physical treatment group included patients aged 4.0–17.9 years who initiated obesity treatment between 2018-2019, referred to a clinic using the digital Evira treatment tool (Evira AB, Stockholm, Sweden). Evira consists of a body scale that does not display digits for daily home-weighing. The measurements are transferred via Bluetooth to a mobile application for the family and a web-based care portal for the clinical staff to enable efficient communication. Body mass index (BMI) Z-score is graphically visualized as a dynamically weighted moving average, reducing the emphasis on individual measurements. This is presented in relation to an individualized weight loss target area set by healthcare staff. The lifestyle support focused on empowering parents to take an active role in the treatment, avoiding specific advice but discussing alternatives and strategies for handling challenges in every-day life. Families were guided to make their own choices and were instructed to adjust eating habits to stay within the individualized weight loss target area and act on deviations. Weekly communication occurred via the clinic’s care portal and the mobile app for families, with additional support.

Since children and adolescents with obesity have an altered height growth pattern [8], the longitudinal growth is estimated accordingly. The standard treatment group consisted of children receiving conventional obesity treatment during a comparable treatment initiation period (October 1st, 2017, to January 1st, 2019). These patients were matched by age ( ± 91 days) and sex, and were randomly selected from the Swedish Childhood Obesity Treatment Register (BORIS) [9] in a 3:1 ratio.

In compliance with Swedish regulations, families received both verbal and written information regarding data collection. Children’s anthropometric data were recorded by healthcare providers during treatment visits. An opt-out approach was used. The trial was ethically approved by the Stockholm Ethics Committee in Sweden (No. 2018/ 1413–31, and amendment 2023-00084-02) and registered in Clinicaltrials.gov ID: NCT06434259.

Treatment outcome was calculated as the change in BMI Z-score from treatment initiation to years 1–3, using the International Obesity Task Force (IOTF) criteria [10]. Annual data included visits with height and weight measurements ±3 months from the anticipated annual follow-up years 1 and 2, and ±6 months for year 3. The non-retention rate excluded individuals who terminated treatment due to obesity remission or being over 18 years of age. The incidence of obesity remission (no obesity according to the IOTF criteria), was calculated. Diagnosis and symptoms of eating disorders were retrieved from patient records in the digi-physical treatment group. All 107 patients were reviewed throughout the entire treatment period and for one additional year.

Descriptive characteristics are presented as proportions, mean and standard deviation (SD), or median and quartiles (Q1, Q3). Analyses were conducted according to intention-to-treat principle, using mixed models to handle missing data without imputation. Change in BMI Z-score over three years was assessed with a mixed model, with random intercept for individual id. Obesity remission incidence rate ratio was estimated using Poisson regression. Both regression models were adjusted for sex, age category ( < 12 years vs. ≥12 years), and degree of obesity (class I vs. class II, corresponding to adult BMI of 30 vs. 35 kg/m^2^) [10] at treatment initiation. Non-retention was estimated using Kaplan-Meier method, with the *p*-value for year 3 retrieved from Poisson regression. Average annual weight measurement frequency was estimated with mixed model, with random intercept for individual id. The analyses were performed in SAS statistical software (version 9.4, Cary, North Carolina).

## Results

A total of 428 patients (67% males) were included, with a median age of 11.5 (Q1: 9.0, Q3: 13.8) years. Among these, 107 received digi-physical treatment with the Evira tool, and 321 received standard treatment. Their respective mean (SD) BMI Z-score at treatment initiation was 2.81 (0.36) vs. 2.77 (0.38), *p* = 0.38. At the three-year follow-up, 50 patients in the digi-physical treatment group were still engaged in treatment, compared with 109 in the standard treatment group, see Supplementary Fig. 1.

**Fig. 1:**
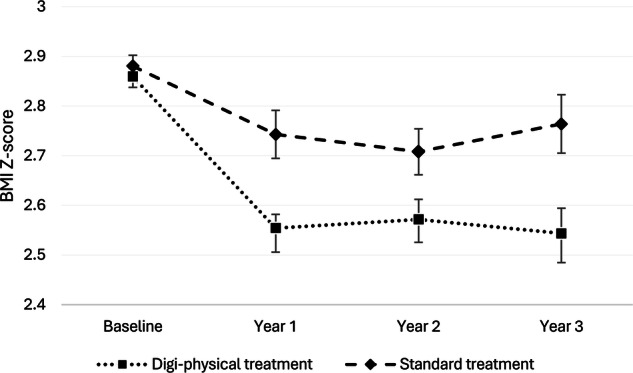
Response to treatment. Estimated BMI Z-score over three years of obesity treatment divided by group. The dotted line represents the digi-physical treatment group, and the dashed line represents the standard treatment group. Error bars are standard error. The regression model was adjusted for sex, age and degree of obesity at treatment initiation.

Following three years of continuous treatment, patients receiving digi-physical treatment exhibited a significantly greater reduction in degree of obesity than the standard treatment group; adjusted average (95% Cl) change in BMI Z-score -0.29 (–0.40 to –0.18) vs. –0.12 (–0.21 to –0.03), *p* = 0.02, as illustrated in Fig. 1. Unadjusted and adjusted estimated average change in BMI Z-score for each year of follow-up are provided in Supplementary Table 1.

The proportion obtaining obesity remission during the three-year follow-up was 31.8% (*n* = 34) in the digi-physical treatment group and 18.7% (*n* = 60) in the standard treatment group, *p* = 0.0046. Greater obesity remission rate was observed in the digi-physical treatment group compared to the standard treatment group, incidence rate ratio (IRR): 1.76 (1.15–2.68), *p* = 0.009. Unadjusted IRR are provided in Supplementary Table 2.

**Fig. 2:**
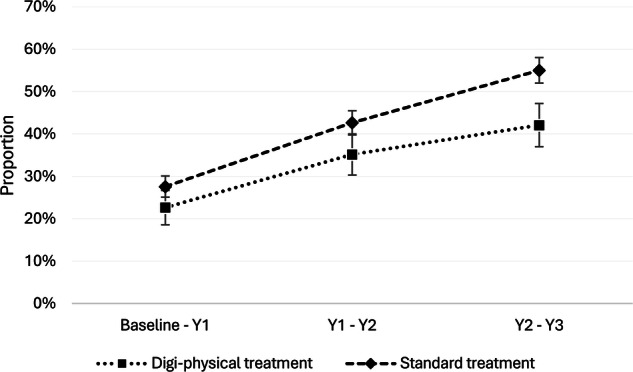
Patients not remaining in treatment. Kaplan-Meier method used to estimate the proportion of non-retention (patient’s wish to terminate treatment or health care’s inability to offer treatment) during the three-year follow-up divided by group. The dotted line represents the digi-physical treatment group, and the dashed line represents the standard treatment group. Error bars are standard error.

Non-retention by year 3 was 42% in the digi-physical treatment group and 55% in the standard treatment group, *p* = 0.0002, Fig. 2. Estimates for non-retention for each year of follow-up are presented in Supplementary Table 3. Compared to individuals without data at year 3, those with data at year 3 included a higher proportion of males and were on average younger, although the pattern was slightly different between the groups, Supplementary Table 4.

Average (95% CI) numbers of home-weighings per week declined during the study period from 3.9 (3.7 to 4.3) during year 1, 3.2 (2.8 to 3.7) year 2 and 2.9 (2.4 to 3.4) year 3.

During follow-up, three patients in the digi-physical treatment group exhibited symptoms of disordered eating behavior. One was recorded with loss of control during the second year of treatment and two were recorded with anxiety in relation to eating one year after the trial period. All three had ongoing specialist psychiatric care for other reasons, such as mental health illness or trauma. None received an eating disorder diagnosis.

Among adolescents, 37% (*n* = 19) in the digi-physical treatment group and 22% (*n* = 31) in standard treatment group had three-year follow-up data. Their corresponding change in BMI Z-score after three years of treatment was -0.38 (–0.59 to –0.17) and +0.02 (–0.14 to +0.18), respectively, *p* = 0.012. Obesity remission rate favored the digi-physical treatment group, yet not statistically significant, IRR (95% CI) = 1.83 (0.92 to 3.64), *p* = 0.083, possibly due to limited power (post hoc β = 0.46). Unadjusted IRR are provided in Supplementary Table 2.

## Discussion

To our knowledge, this is the first trial providing long-term evidence that digi-physical treatment for children and adolescents with obesity not only is feasible but also yields superior treatment-related outcomes compared to standard treatment. After three years, patients in the digi-physical treatment group experienced more than twice the mean relative weight loss (–0.29 vs. –0.12 BMI Z-score units), with particularly notable results in adolescents (–0.38 vs. 0.02 BMI Z-score units). Additionally, a higher proportion achieved obesity remission (32% vs. 19%).

The frequency of clinical visits is crucial for treatment outcomes, with the most effective treatments involving 26 or more hours of contact [3]. Achieving this frequency is challenging for both families and healthcare providers. This study suggests that integrating a digital treatment tool with physical visits is an effective way to achieve the necessary intensity.

After three years, the digi-physical treatment group had a higher proportion of patients remaining in treatment, adressing a general concern about long-term sustainibility [4]. Nevertheless, the non-retention rate was high in both groups (digi-physical treatment 42% and standard treatment 55%), reflecting a well-established challenge in obesity treatment [11], possibly further aggravated by the Covid-19 pandemic.

Weight regain, commonly observed in obesity treatment [12], was observed in the standard treatment group but not in the digi-physical treatment group, indicating the potential of the digital tool in sustaining initial weight loss.

The Evira digital treatment tool is designed to enhance parental empowerment, diverging from traditional approaches that primarily focus on advising about eating habits and physical activity, cornerstones in conventional treatment [13, 14]. Recognizing that parental knowledge of nutrition is generally adequate [15, 16], the health care team instead encourages the parents to devise personalized strategies for reducing total energy intake, tailored to their unique living conditions. The frequent home-weighing, coupled with height growth predictions and calculated BMI Z-score changes visualized in the app, provide the parents with direct feedback whether their implemented changes are adequate for achieving significant relative weight loss.

While dietary restraint and focusing on weight are considered risk factors for developing eating disorders, they remain components of obesity treatment [17]. In this trial, three patients exhibited symptoms of disordered eating behavior in concurrence with other mental health conditions. It is impossible to conclude if this number is lower or higher than anticipated due to the limited number of individuals and the pragmatic design. Nevertheless, current evidence supports that neither daily home-weighing from age 18 nor structured pediatric obesity treatment increases the risk of anxiety, depression, or eating disorders [17, 18].

Limitations of this trial include reliance on data from a single clinic with a limited number of patients. Nevertheless, the digi-physical population heterogeneity regarding previously received treatment, neuropsychiatric conditions, and migration background [6] improves generalizability. Furthermore, given the pragmatic trial design, standardized data collection of lifestyle factors, such as stress and sleep, was not conducted. Additionally, the standard treatment group lacked data on eating disorder diagnoses or symptoms, preventing comparisons between groups. Lastly, although the follow-up period was relatively long, it is important to note that the results are based on data from a single clinic, which may limit the generalizability. Therefore, further studies involving multiple clinics, preferably in diverse settings, are needed to validate and expand upon these findings.

As Evira seems to reduce weight regain, future studies could also focus on combining different approaches to optimize treatment response. Integrating Evira with pharmacological and bariatric treatments for obesity could facilitate the monitoring of weight development and early identification when additional support is needed.

In conclusion, long-term digi-physical treatment with frequent home-weighings demonstrates a greater reduction in the degree of obesity, a higher obesity remission rate, and lower non-retention in children aged 4–17 years compared to standard treatment.

## Supplementary information

Supplementary material

## Data Availability

The data that support the findings of this study are available from Evira AB but restrictions apply to the availability of these data, which were used under license for the current study, and so are not publicly available. Data are however available from the authors upon reasonable request and with permission of Evira AB.

## References

[1] Marcus C, Danielsson P, Hagman E. Pediatric obesity-Long-term consequences and effect of weight loss. J Intern Med. 2022;292:870–91. 35883220 10.1111/joim.13547 PMC9805112

[2] Putri RR, Danielsson P, Ekstrom N, Ericsson A, Lindberg L, Marcus C, et al. Effect of pediatric obesity treatment on long-term health. JAMA Pediatr. 2025;179:302–9. 10.1001/jamapediatrics.2024.5552 PMC11877215 39836390

[3] O’Connor EA, Evans CV, Burda BU, Walsh ES, Eder M, Lozano P. Screening for obesity and intervention for weight management in children and adolescents: evidence report and systematic review for the US preventive services task force. J Am Med Assoc. 2017;317:2427–44.10.1001/jama.2017.033228632873

[4] Azevedo LB, Stephenson J, Ells L, Adu-Ntiamoah S, DeSmet A, Giles EL, et al. The effectiveness of e-health interventions for the treatment of overweight or obesity in children and adolescents: A systematic review and meta-analysis. Obes Rev. 2022;23:e13373. 34747118 10.1111/obr.13373

[5] Johansson L, Hagman E, Danielsson P. A novel interactive mobile health support system for pediatric obesity treatment: a randomized controlled feasibility trial. BMC Pediatr. 2020;20:447. 32967638 10.1186/s12887-020-02338-9 PMC7513491

[6] Hagman E, Johansson L, Kollin C, Marcus E, Drangel A, Marcus L, et al. Effect of an interactive mobile health support system and daily weight measurements for pediatric obesity treatment, a 1-year pragmatical clinical trial. Int J Obes (Lond). 2022;46:1527–33. 35641569 10.1038/s41366-022-01146-8 PMC9314258

[7] Miller WR, Rollnick S. *Motivational interviewing : helping people change and grow*, Fourth edition. edn The Guilford Press: New York, 2023.

[8] Putri RR, Danielsson P, Marcus C, Hagman E. Height and growth velocity in children and adolescents undergoing obesity treatment: a prospective cohort study. J Clin Endocrinol Metab. 2023;109:e314–e320. 37453086 10.1210/clinem/dgad419 PMC10735311

[9] Hagman E, Danielsson P, Lindberg L, Marcus C. Committee BS. Paediatric obesity treatment during 14 years in Sweden: Lessons from the Swedish Childhood Obesity Treatment Register-BORIS. Pediatr Obes. 2020;15:e12626. 32074662 10.1111/ijpo.12626

[10] Cole TJ, Lobstein T. Extended international (IOTF) body mass index cut-offs for thinness, overweight and obesity. Pediatr Obes. 2012;7:284–94. 22715120 10.1111/j.2047-6310.2012.00064.x

[11] Skelton JA, Beech BM. Attrition in paediatric weight management: a review of the literature and new directions. Obes Rev : Off J Int Assoc Study Obes. 2011;12:e273–81. 10.1111/j.1467-789X.2010.00803.x PMC3079805 20880126

[12] Vermeiren E, Bruyndonckx L, De Winter B, Verhulst S, Van Eyck A, Van Hoorenbeeck K. The effect of weight regain on cardiometabolic health in children with obesity: a systematic review of clinical studies. Nutr Metab Cardiovascular Dis. 2021;31:2575–86. 10.1016/j.numecd.2021.05.020 34172320

[13] Hampl SE, Hassink SG, Skinner AC, Armstrong SC, Barlow SE, Bolling CF, et al. Clinical practice guideline for the evaluation and treatment of children and adolescents with obesity. Pediatrics. 2023;151:e2022060640. 36622115 10.1542/peds.2022-060640

[14] Lister NB, Baur LA, Felix JF, Hill AJ, Marcus C, Reinehr T, et al. Child and adolescent obesity. Nat Rev Dis Prim. 2023;9:24. 37202378 10.1038/s41572-023-00435-4

[15] Lee H, Oldewage-Theron W, Dawson JA. Effects of a theory-based, multicomponent ehealth intervention for obesity prevention in young children from low-income families: a pilot randomized controlled study. Nutrients. 2023;15:2296. 37242179 10.3390/nu15102296 PMC10222385

[16] Mazurkiewicz A, Raczkowska E. The connection between knowledge and the nutritional behaviour of parents and the occurrence of overweight and obesity among preschool children-a pilot study. Nutrients. 2024;16:174. 38202003 10.3390/nu16010174 PMC10780658

[17] Lister NB, Baur LA, Paxton SJ, Jebeile H. Contextualising eating disorder concerns for paediatric obesity treatment. Curr Obes Rep. 2021;10:322–31. 33970441 10.1007/s13679-021-00440-2

[18] Gorin AA, Gokee LaRose J, Espeland MA, Tate DF, Jelalian E, Robichaud E, et al. Eating pathology and psychological outcomes in young adults in self-regulation interventions using daily self-weighing. Health Psychol. 2019;38:143–50. 30550313 10.1037/hea0000689 PMC6447368

