# Supplementary material for: Long-term results of a digital treatment tool as an add-on to pediatric obesity lifestyle treatment: a 3-year pragmatic clinical trial
Source: Int J Obes (Lond). 2025 Mar 12;49(5):973–6. doi: 10.1038/s41366-025-01738-0 (PMC12095034; doi:10.1038/s41366-025-01738-0)
Supplement: Supplementary file 1 — Supplementary material [file 41366_2025_1738_MOESM1_ESM.docx]

**Supplementary material**

Long-term results of a digital treatment tool as an add-on to pediatric obesity lifestyle treatment: A 3-year pragmatic clinical trial

Emilia Hagman E^1^, Louise Lindberg^1,2^, Resthie R. Putri^1^, Andreas Drangel ^2^, Claude Marcus^1,2^, Pernilla Danielsson^1^

^1^Department of Clinical Science, Intervention and Technology, Division of Pediatrics, Karolinska Institutet, Sweden ^2^Evira AB, Triewaldsgränd 2, S-111 29 Stockholm, Sweden.

**Corresponding author:** Pernilla Danielsson, [pernilla.danielsson-liljeqvist@regionstockholm.se](mailto:pernilla.danielsson-liljeqvist@regionstockholm.se)

STANDARD CARE

START OF TREATMENT

n=321

YEAR 1

Patients in treatment, n=231

Missing data at year 1, n=33

Included in analyses, n=198

YEAR 2

Patients in treatment, n=160

Missing data at year 2, n=29

Included in analyses, n=131

YEAR 3

Patients in treatment, n=109

Included in analyses, n=109

Obesity remission, n=0

Over 18 years, n=2

Non-retention, n=88

Obesity remission, n=20

Over 18 years, n=9

Non-retention, n=42

Obesity remission, n=14

Over 18 years, n=7

Non-retention, n= 30

EVIRA

START OF TREATMENT

n=107

YEAR 1

Patients in treatment, n=82

Missing data at year 1, n=4

Included in analyses, n=78

YEAR 2

Patients in treatment, n=57

Missing data at year 2, n=3

Included in analyses, n=54

YEAR 3

Patients in treatment, n=50

Included in analyses, n=50

Obesity remission, n=0

Over 18 years, n=1

Non-retention, n=24

Obesity remission, n=9

Over 18 years, n=5

Non-retention, n=11

Obesity remission, n=1

Over 18 years, n=0

Non-retention, n=6

**Supplementary Figure 1.** Flowchart of patients over three years of treatment. “Non-retention” reflects patient’s wish to terminate treatment or health care’s inability to offer treatment.

| **Supplementary Table 1.** Estimated average change in BMI Z-score and associated 95% confidence intervals for each year of follow-up from mixed model. | | | | |
| --- | --- | --- | --- | --- |
|  | Unadjusted estimates | | Adjusted estimates^1^ | |
|  | Digi-physical treatment | Standard treatment | Digi-physical treatment | Standard treatment |
| Year 1 | -0.28 (-0.37 to -0.19) | -0.12 (-0.17 to -0.08) | -0.28 (-0.38 to -0.19) | -0.13 (-0.17 to -0.08) |
| Year 2 | -0.28 (-0.37 to -0.19) | -0.18 (-0.25 to -0.11) | -0.27 (-0.37 to -0.18) | -0.18 (-0.24 to -0.11) |
| Year 3 | -0.29 (-0.40 to -0.18) | -0.13 (-0.22 to -0.03) | -0.29 (-0.40 to -0.18) | -0.12 (-0.21 to -0.03) |
| ^1^ Adjusted for sex, age category (<12 years vs. ≥12 years), and degree of obesity (class I vs. class II). | | | | |

| **Supplementary Table 2.** Unadjusted incidence rate ratio (IRR) and associated 95% confidence intervals (CI) for obesity remission. | |
| --- | --- |
| Digi-physical treatment vs Standard Care | IRR (95% CI) |
| All | 1.56 (1.02 - 2.38) |
| Adolescents only | 1.70 (0.86 - 3.36) |

| **Supplementary Table 3.** Estimates for non-retention and associated 95% confidence intervals for each year of follow-up from Kaplan-Meier. P-values were obtained from Poisson regression analysis. | | | |
| --- | --- | --- | --- |
|  | Digi-physical treatment | Standard treatment | p between groups |
| Year 1 | 0.23 (0.16 to 0.32) | 0.27 (0.23 to 0.33) | 0.28 |
| Year 2 | 0.35 (0.27 to 0.46) | 0.43 (0.37 to 0.48) | 0.051 |
| Year 3 | 0.42 (0.33 to 0.53) | 0.55 (0.49 to 0.61) | 0.0002 |

| **Supplementary Table 4.** Baseline characteristics of participants with and without 3-year follow-up data. | | | | | | | | | |
| --- | --- | --- | --- | --- | --- | --- | --- | --- | --- |
|  | All | | | Digi-physical treatment | | | Standard treatment | | |
|  | With data  (n = 159) | Without data  (n = 269) | p | With data  (n = 50) | Without data  (n = 57) | p | With data  (n = 109) | Without data  (n = 212) | p |
| Sex, Females / Males (%) | 23.9 / 76.1 | 37.9 / 62.1 | 0.0028 | 24.0 / 76.0 | 40.4 / 59.6 | 0.072 | 29.9 / 76.1 | 37.2 / 62.7 | 0.015 |
| Age at treatment initiation, mean (SD) | 10.5 (2.7) | 12.1 (3.2) | <.0001 | 11.2 (2.6) | 15.5 (3.4) | 0.030 | 10.1 (2.6) | 12.0 (3.1) | <.0001 |
| BMI Z-score at treatment initiation, mean (SD) | 2.79 (0.38) | 2.78 (0.37) | 0.80 | 2.79 (0.33) | 2.82 (0.39) | 0.68 | 2.78 (0.40) | 2.77 (0.37) | 0.68 |
| Differences in proportions were assessed using Chi-squared test, and t-tests were applied for continuous variables. | | | | | | | | | |
